# Hearing loss, hearing aid use, and performance on the Montreal cognitive assessment (MoCA): findings from the HUNT study in Norway

**DOI:** 10.3389/fnins.2023.1327759

**Published:** 2024-01-08

**Authors:** Shahram Moradi, Bo Engdahl, Aud Johannessen, Geir Selbæk, Lisa Aarhus, Gro Gade Haanes

**Affiliations:** ^1^Research Group for Disability and Inclusion, Faculty of Health and Social Sciences, Department of Health, Social and Welfare Studies, University of South-Eastern Norway Campus Porsgrunn, Porsgrunn, Norway; ^2^Research Group for Health Promotion in Settings, Department of Health, Social and Welfare Studies, University of South-Eastern Norway, Tønsberg, Norway; ^3^Department of Physical Health and Ageing, Norwegian Institute of Public Health, Oslo, Norway; ^4^Faculty of Health and Social Sciences, Department of Health, Social and Welfare Studies, University of South-Eastern Norway Campus Vestfold, Horten, Norway; ^5^Norwegian National Centre for Ageing and Health, Tønsberg, Norway; ^6^Institute of Clinical Medicine, Faculty of Medicine, University of Oslo, Oslo, Norway; ^7^Geriatric Department, Oslo University Hospital, Oslo, Norway; ^8^Department of Occupational Medicine and Epidemiology, National Institute of Occupational Health, Oslo, Norway; ^9^Medical Department, Diakonhjemmet Hospital, Oslo, Norway; ^10^USN Research Group of Older Peoples’ Health, University of South-Eastern Norway Department of Nursing and Health Sciences, Faculty of Health and Social Sciences, University of South-Eastern Norway, Drammen, Norway

**Keywords:** cognitive function, cognitive decline, hearing loss, hearing aid use, Montreal Cognitive Assessment (MoCA)

## Abstract

**Purpose:**

To evaluate the associations between hearing status and hearing aid use and performance on the Montreal Cognitive Assessment (MoCA) in older adults in a cross-sectional study in Norway.

**Methods:**

This study utilized data from the fourth wave of the Trøndelag Health Study (HUNT4, 2017–2019). Hearing thresholds at frequencies of 0.5, 1, 2, and 4 kHz (or PTA4) in the better hearing ear were used to determine participants’ hearing status [normal hearing (PTA4 hearing threshold, ≤ 15 dB), or slight (PTA4, 16–25 dB), mild (PTA4, 26–40 dB), moderate (PTA4, 41–55 dB), or severe (PTA4, ≥ 56 dB) hearing loss]. Both standard scoring and alternate MoCA scoring for people with hearing loss (deleting MoCA items that rely on auditory function) were used in data analysis. The analysis was adjusted for the confounders age, sex, education, and health covariates.

**Results:**

The pattern of results for the alternate scoring was similar to that for standard scoring. Compared with the normal-hearing group, only individuals with moderate or severe hearing loss performed worse in the MoCA. In addition, people with slight hearing loss performed better in the MoCA than those with moderate or severe hearing loss. Within the hearing loss group, hearing aid use was associated with better performance in the MoCA. No interaction was observed between hearing aid use and participants’ hearing status with performance on the MoCA test.

**Conclusion:**

While hearing loss was associated with poorer performance in the MoCA, hearing aid use was found to be associated with better performance in the MoCA. Future randomized control trials are needed to further examine the efficacy of hearing aid use on the MoCA performance. When compared with standard scoring, the alternate MoCA scoring had no effect on the pattern of results.

## Introduction

Cognitive function is a broad and multidimensional term that includes domains such as concentration and attention, psychomotor efficiency, learning and memory, visuospatial abilities, verbal fluency, problem-solving, manual dexterity, and mental flexibility (Ryan et al., 1987). The extent to which hearing loss affects cognitive function has received increasing interest in the last decade. Loughrey et al. (2018), in their review and meta-analysis, concluded that hearing loss is a potential risk factor for cognitive impairment and dementia. Recently, Marinelli et al. (2022), in a prospective population-based study, showed that hearing loss was linked to poor cognitive function over time. Currently, hearing aids are the main treatment option; these amplify the sound for better hearing. Yeo et al. (2023) in a literature review revealed that the use of hearing aids and cochlear implants by people with hearing difficulties was associated with a 9% decrease in the risk of long-term cognitive decline and a 3% improvement in cognitive tests that measure global cognitive function in the short term. However, there are other literature review studies that debate the positive effect of hearing aid use on cognitive function (e.g., Sanders et al., 2021; Dawes and Völter, 2023). Dawes and Völter (2023), for example, noted that fewer than half of prior studies reported better performance in cognitive function following hearing aid use. In addition, most prior studies had short duration (from few weeks up to 18 months). As cognitive decline is a gradual process, trials with longer duration of interventions are needed. Thus, it is unclear whether hearing aid use can safeguard against cognitive decline in people with hearing loss.

The Montreal Cognitive Assessment (MoCA) is a brief cognitive screening measure designed to evaluate the cognitive status of individuals across various cognitive domains such as: attention/concentration, memory, executive and visuospatial skills, spatial orientation, mental dexterity, and language fluency (Nasreddine et al., 2005). The MoCA has been used to investigate the extent to which hearing loss affects cognitive function. Current literature has shown that hearing loss is associated with poor scores in the MoCA (e.g., Wang et al., 2022; Fu et al., 2023).

To the best of our knowledge, few studies investigated the effect of hearing aid use on MoCA performance (e.g., Saunders et al., 2018; Cuoco et al., 2021; Dawes and Völter, 2023). In Saunders and colleagues’ study (2018), participants with hearing loss were tested once under unaided conditions and once under aided listening conditions when performing the MoCA. Results showed that the amplification of sounds by a hearing aid had no effect on participants’ MoCA performance. Cuoco et al. (2021), in a prospective study, compared people with hearing loss who tolerated using hearing aids versus those who did not tolerate using hearing aids according to their performance in a neuropsychological battery that included the MoCA. After 6 months of using hearing aids, the results showed no significant difference between hearing-aid users and non-hearing-aid users in terms of MoCA performance. Glick and Sharma (2020), however, in their study of people with mild to moderate hearing loss showed that using a hearing aid for 6 months was associated with better speech-in-noise perception and improved cognitive function (measured with the MoCA). Their study also showed that hearing aid use can reverse cortical changes caused by hearing loss. Vasil et al. (2021) studied whether cochlear implantation can lead to better performance on the MoCA after 6 months of cochlear implantation. The results showed that cochlear implantation improved the performance in the MoCA test (that was administered audiovisually). The authors reasoned that this improvement was mainly due to enhanced performance in the “delayed recall” aspect of the test that relies on auditory function.

Dupuis et al. (2015) highlighted that, as some items of the MoCA rely on sensory functioning (auditory and vision), consequently performance in the test might be highly dependent on the sensory function of individuals, especially in older adults. The study also found that individuals with normal sensory acuity performed better in the MoCA than those with sensory loss, despite modifying the scores of the MoCA test for people with sensory loss. These findings led to the development of a version of the MoCA for people with hearing loss (MoCA-H) (Dawes et al., 2023; Völter et al., 2023).

However, some studies have shown that the test administration conditions (auditory or visual presentations of MoCA items) have no effect on the MoCA performance. For example, Shen et al. (2020) examined the extent to which the test administration conditions (auditory amplified, auditory unamplified, and visual) affected the MoCA performance of older adults with hearing loss. Their results showed that neither amplification nor test modality influenced participants’ performance in the MoCA. Parada et al. (2020) compared the MoCA version for people with hearing loss with the standard version of the MoCA among postlingually deafened cochlear implant users. No significant difference was observed in participants’ performance between the HI-MoCA and standard MoCA.

Using a larger sample than prior studies, this cross-sectional study sought to examine the associations between hearing loss and hearing aid use and performance in the MoCA, after controlling for confounders including age, education, sex, and health covariates, using data from the HUNT4 study in Norway. In addition, we compared the pattern of results acquired from standard scoring with those from alternate MoCA scoring for people with hearing loss (as developed and used by Dupuis et al., 2015) in order to examine how MoCA scoring affects the evaluation of cognitive function in older adults with various hearing abilities.

## Materials and methods

### Participants

Participants in this study were those who took part in both HUNT4 Hearing and HUNT4 70+ studies, as parts of the HUNT4 study conducted in Nord-Trøndelag County in Norway (Åsvold et al., 2023). In the HUNT4 Hearing study, all inhabitants in the six larger municipalities (Levanger, Stjørdal, Steinkjer, Verdal, Nærøy, Namsos), representing about two thirds of the Nord-Trøndelag region who were aged ≥20 years were invited to undertake hearing screening using objective and subjective measures. Around 43% took part in HUNT4 Hearing study. Detailed information about the HUNT4 Hearing study is available in Engdahl et al. (2021).

In the HUNT4 70+ study, all inhabitants aged ≥70 years in the Nord-Trøndelag County, a total of 19,403 individuals, born between 1931 and 1949, were invited for clinical assessment of cognitive functioning and dementia. Around 51% took part in the HUNT4 70+ study. Detailed information about the HUNT4 70+ study is available in Gjøra et al. (2021).

The present study includes 5,364 subjects > = 70 years of age participating in both HUNT4 70+ and HUNT4 Hearing. Participants provided informed consent for their participation in both HUNT4 Hearing and HUNT4 70+ studies. The data collection for both HUNT4 Hearing and HUNT4 70+ took place between September 2017 and March 2019. The regional committee for medical and health research ethics approved the study (ID Number: 23178 Hørsel).

### Hearing status

Detailed information about the hearing screening of participants in the HUNT4 Hearing study is available in Engdahl et al. (2021). Trained health teams collected the audiological data in the HUNT4 Hearing research; each team consisted of a trained audiologist and two trained assistants. First, a questionnaire was employed to check for tinnitus, subjective hearing loss, and hearing aid use. Then, each participant underwent pure tone audiometry and otoscopy. Pure tone audiometry was carried out using Interacoustics audiometers (type AD629) in semiportable, dismountable sound booths (IAC Moduline System; 102 mm thick, 1,450 × 1,450 × 2,100 mm^3^).

In this study, the hearing status of participants was defined based on a pure tone average of four frequencies (0.5, 1, 2, and 4 kHz, or PTA4) for the better hearing ear, and categorized as: normal hearing (PTA4 hearing threshold, ≤ 15 dB); slight hearing loss (PTA4, 16–25 dB), mild hearing loss (PTA4, 26–40 dB), moderate hearing loss (PTA4, 41–55 dB), and severe hearing loss (PTA4, ≥ 56 dB).

### Montreal cognitive assessment (MoCA)

In the HUNT4 70+ study, the Norwegian version of the MoCA (version 7.1) was used to evaluate participants’ cognitive function. The MoCA assesses cognitive function in different cognitive domains, including visuospatial or executive functioning, animal naming, memory, attention, language, abstraction, delayed recall, and orientation. Scores on each cognitive domain are combined to calculate the total sum score. The MoCA score ranges from 0 to 30, whereby a higher score reflects better cognitive function. The education adjustment (1 additional point for individuals with 12 years of education or less) was not applied. The participants were tested by trained health personnel at a field station, in their nursing homes, or in their own homes. The health personnel were trained rigorously before data collection; the importance of participants being able to see and/or hear the items in the MoCA items was emphasized. The participants were asked to wear their hearing aids and glasses during testing if they felt it was necessary.

We used both the standard scoring procedure (score range of 0–30) and the alternate scoring system (score range of 0–20) for people with hearing loss (the alternate version) developed by Dupuis et al. (2015) for data analysis. Dupuis et al. (2015) removed four MoCA items (language repetition, attention to letters, digit span, and delayed recall) that rely heavily on hearing function for task performance (i.e., repeating or remembering stimuli). The alternate scoring method allows us to investigate the notion that the worse performance of people with hearing loss in the MoCA is mainly due to poor hearing rather than reduced cognitive function.

### Statistical analysis

SPSS statistical software (version 29) was used for data analysis. Analysis of variance (ANOVA) was chosen in order to assess the main effects of hearing status and hearing aid use and interaction between hearing status and hearing aid use on MoCA scores (for both standard scoring and alternate scoring procedures). Pairwise comparisons were computed using the Bonferroni method, to determine which pair of groups exhibited significant differences.

Hearing status and hearing aid use were considered as independent variables and MoCA scores as dependent variable. Age, sex, and education were considered as confounding variables in this study. Age was categorized into five age groups, (70–74, 75–79, 80–84, 85–89, and 90+ years) and education was divided into three educational levels (primary, secondary, and tertiary). These variables were treated as fixed factors in the analysis. In addition, all analyses were controlled for covariates like a heart attack, heart failure, cancer, stroke, diabetes, smoking, and hospital admission for head injury. Data regarding confounders and covariates were collected using questionnaires in the HUNT 4. These confounders and covariates were chosen based on prior studies in the literature and factors that affect hearing and cognitive functioning in middle and late life. All confounders and covariates in the analysis were categorical variables that had two or more than two categories. These categorical variables allow us to categorize the participants in classes that made it possible to conduct ANOVA for the purpose of this study.

To address the issue of missing data, listwise deletion was employed. The numbers of individuals with missing data for confounders and covariates are as follows: age, *n* = 0; sex, *n* = 0; education, *n* = 59; heart attack, *n* = 483; heart failure, *n* = 556; cancer, *n* = 439; stroke, *n* = 531; diabetes, *n* = 162; smoking, *n* = 59; and hospital admission for head injury, *n* = 623.

## Results

The basic characteristics of participants, stratified by hearing status, are shown in Table 1. Participants with moderate or severe hearing loss were somewhat older than those with normal hearing or slight hearing loss. In addition, hearing aid use was more common in moderate and severe hearing loss than slight and mild hearing loss. While women constituted two-thirds of participants in the normal hearing group, men constituted two-thirds of participants in the severe hearing loss group.

**Table 1:** Participants’ characteristics stratified by their hearing status (*n* = 5,364).

Hearing status	*n*	Age (mean (SD); years)	Sex (%)	Average PTA4 for best ear (SD)	MoCA (standard scoring) mean (SD)	MoCA (alternate scoring) mean (SD)	Hearing-aid user? (%)	Stroke? (%)	Heart attack? (%)	Hospital admission for head injury? (%)	Diabetes? (%)	Cancer? (%)	Smoking habits (%)	Educational level *(%)
Normal hearing	1,210	74.1 (3.6)	Females: 64 Males: 36	13.1 (6.2)	24.2 (3.5)	17.4 (2.3)	–	Yes (7) No (93)	Yes (9) No (91)	Yes (93) No (6) I do not know (1)	Yes (9) No (91)	Yes (18) No (82)	Never smoked (38) Former occasional smoker (5) Former daily smoker (49) Smoking occasionally (0.1) Daily smoker (7)	Primary (21) Secondary (46) Tertiary (33)
							–							
Slight hearing loss	1,617	75.2 (4.1)	Females: 56 Males: 44	23.5 (6.0)	23.7 (3.6)	17.1 (2.3)	Yes (4) No (96)	Yes (8) No (92)	Yes (10) No (90)	Yes (6) No (93) I do not know (0.5)	Yes (13) No (87)	Yes (21) No (79)	Never smoked (38) Former occasional smoker (5) Former daily smoker (50) Smoking occasionally (0.2) Daily smoker (7)	Primary (26) Secondary (47) Tertiary (28)
Mild hearing loss	1706	77.3 (5.2)	Females: 51 Males: 49	35.7 (7.7)	22.9 (4.2)	16.6 (2.7)	Yes (22) No (78)	Yes (9) No (91)	Yes (11) No (89)	Yes (6) No (93) I do not know (1)	Yes (12) No (88)	Yes (17) No (83)	Never smoked (36) Former occasional smoker (5) Former daily smoker (53) Smoking occasionally (0.5) Daily smoker (6)	Primary (29) Secondary (46) Tertiary (26)
Moderate hearing loss	674	79.9 (5.8)	Females: 40 Males: 60	49.8 (7.8)	21.9 (4.6)	16.1 (3.1)	Yes (62) No (38)	Yes (10) No (90)	Yes (13) No (87)	Yes (7) No (92) I do not know (1)	Yes (14) No (86)	Yes (20) No (80)	Never smoked (37) Former occasional smoker (3) Former daily smoker (54) Smoking occasionally (1) Daily smoker (6)	Primary (30) Secondary (49) Tertiary (22)
Severe hearing loss	157	82.3 (6.1)	Females: 38 Males: 62	68.0 (12.6)	21.1 (5.5)	15.7 (3.6)	Yes (78) No (22)	Yes (11) No (89)	Yes (18) No (82)	Yes (9) No (89) I do not know (2)	Yes (17) No (83)	Yes (24.5) No (75.5)	Never smoked (37) Former occasional smoker (5) Former daily smoker (50) Smoking occasionally (1) Daily smoker (7)	Primary (29) Secondary (48) Tertiary (23)

SD, standard deviation; PTA4, four-frequency pure tone average; MoCA, Montreal Cognitive Assessment. ^*^Educational level was coded into three levels of highest educational levels that were achieved: primary (primary and secondary school), secondary (high school or equivalent), and tertiary (university and/or college).

**Figure 1:**
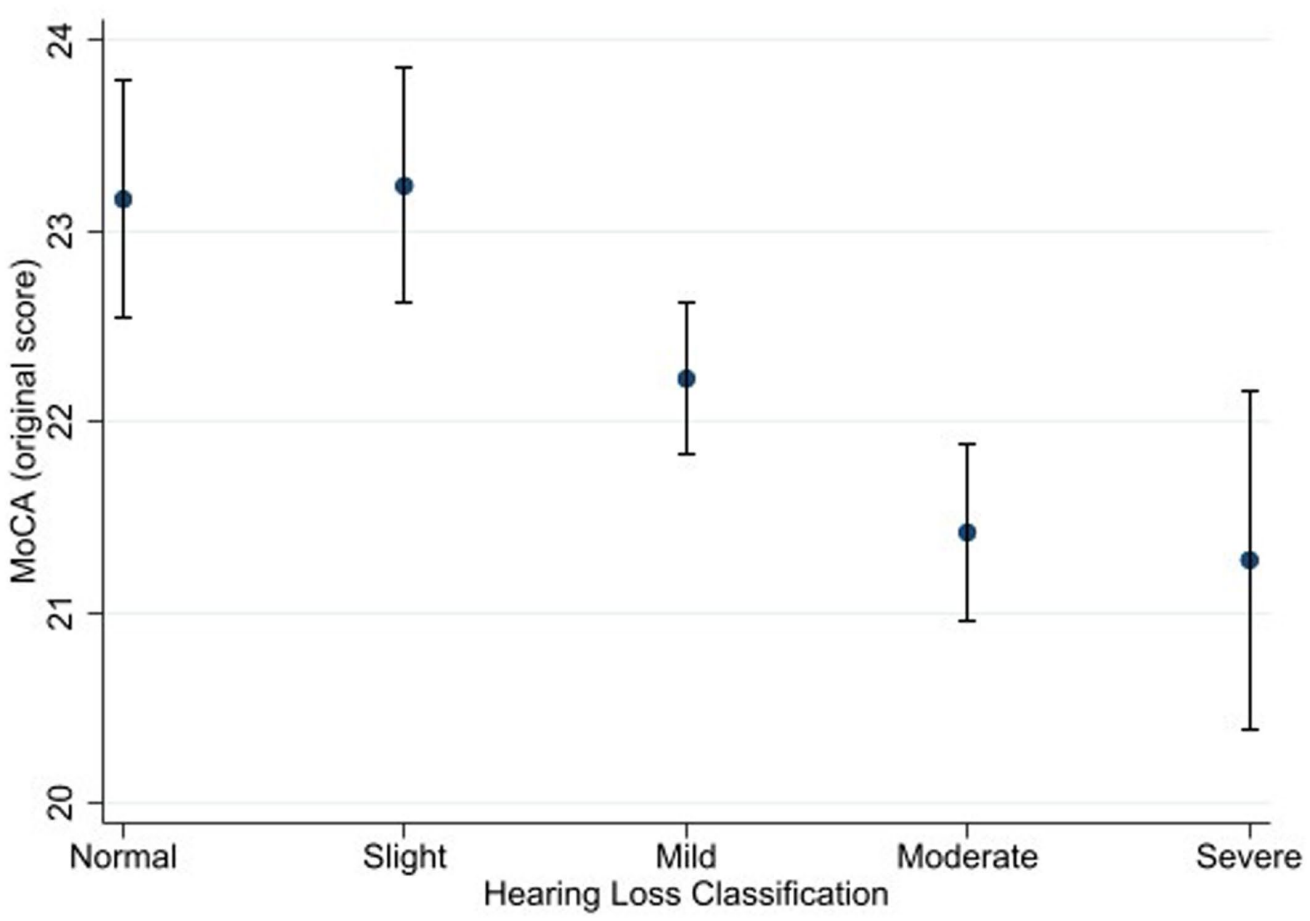
Estimated marginal means of the Montreal Cognitive Assessment (standard scoring) as a function of hearing status. Estimates are averaged over categories of age, education, and sex at the means of the covariates of heart attack, heart failure, cancer, stroke, diabetes, smoking, and hospital admission for head injury. The error bars indicate 95% confidence intervals.

**Figure 2:**
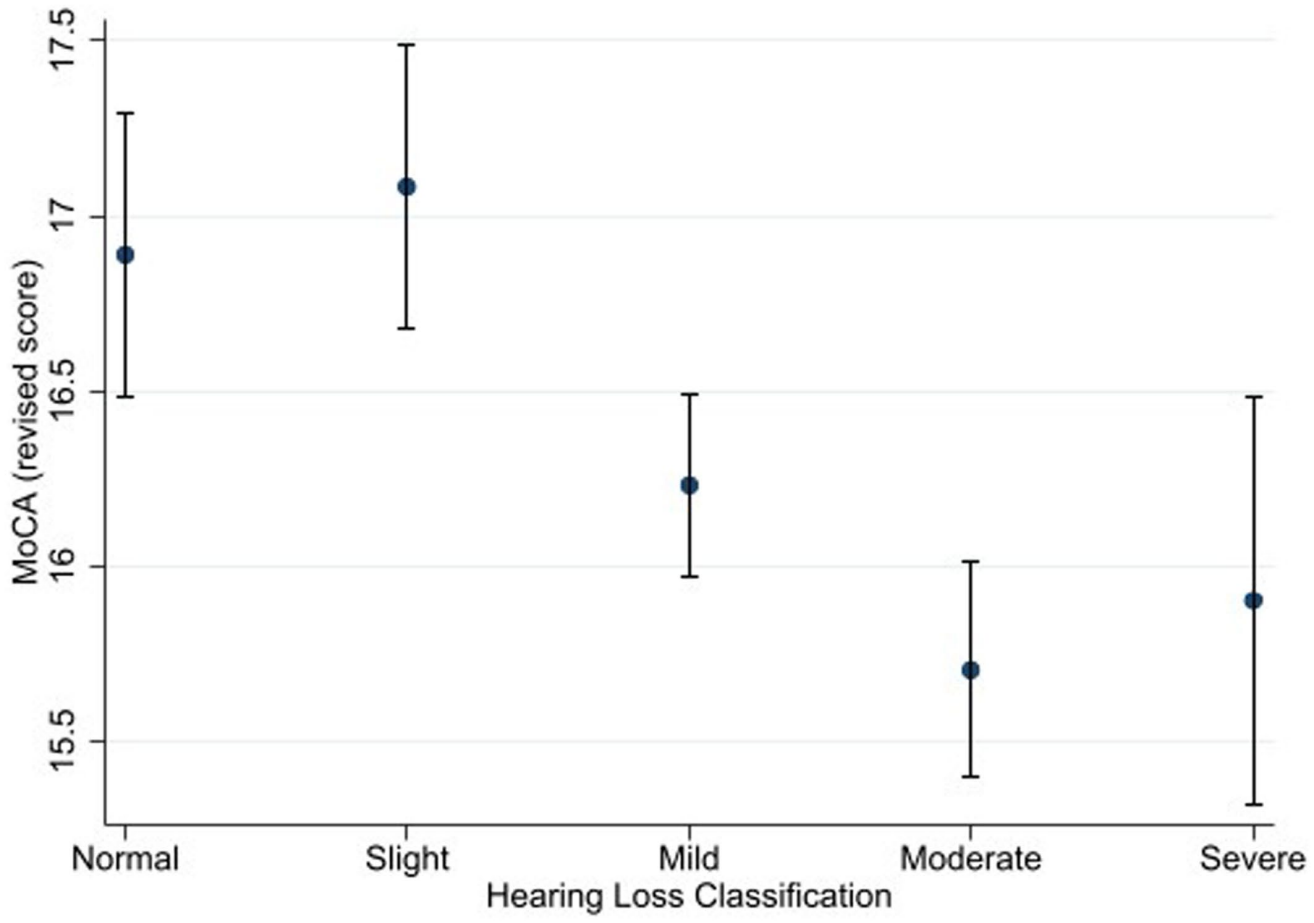
Estimated marginal means of the Montreal Cognitive Assessment (alternate scoring) as a function of hearing status. Estimates are averaged over categories of age, education, and sex at the means of the covariates of heart attack, heart failure, cancer, stroke, diabetes, smoking, and hospital admission for head injury. The error bars indicate 95% confidence intervals.

Figures 1, 2 depict the estimated marginal means for the standard and alternate scoring of the MoCA as a function of participants’ hearing status. Using the standard scoring method, a four-way ANOVA showed a significant main effect for hearing status [*F* (4, 3,506) = 3.21, *p* = 0.012, η^2^ = 0.004]. This main effect indicates that there are differences among different groups in this study with regards to their scores in the MoCA. Bonferroni-adjusted pairwise comparisons revealed that the normal hearing group (M *=* 23.2) achieved a higher score than the moderate (M *=* 21.4) and severe (M *=* 21.3) hearing loss groups. In addition, the slight hearing loss group (M *=* 23.2) achieved a higher score than the moderate and severe hearing loss groups. The difference between the normal hearing loss group and the mild hearing loss group (M *=* 22.2) was marginally insignificant (*p* = 0.067).

Using the alternate scoring procedure, the results showed a significant main effect of hearing status [*F* (4, 3,506) = 4.06, *p* = 0.003, η^2^ = 0.005]. Bonferroni-adjusted pairwise comparisons showed that that the normal hearing group (M *=* 16.9) achieved a higher score than the moderate hearing loss group (M *=* 15.7). The difference between the normal hearing loss group and the severe hearing loss group (M *=* 15.9) was marginally insignificant (*p* = 0.064). The slight hearing loss group (M *=* 17.1) achieved a higher score than the mild (M *=* 16.2), moderate, and severe hearing loss groups.

**Figure 3:**
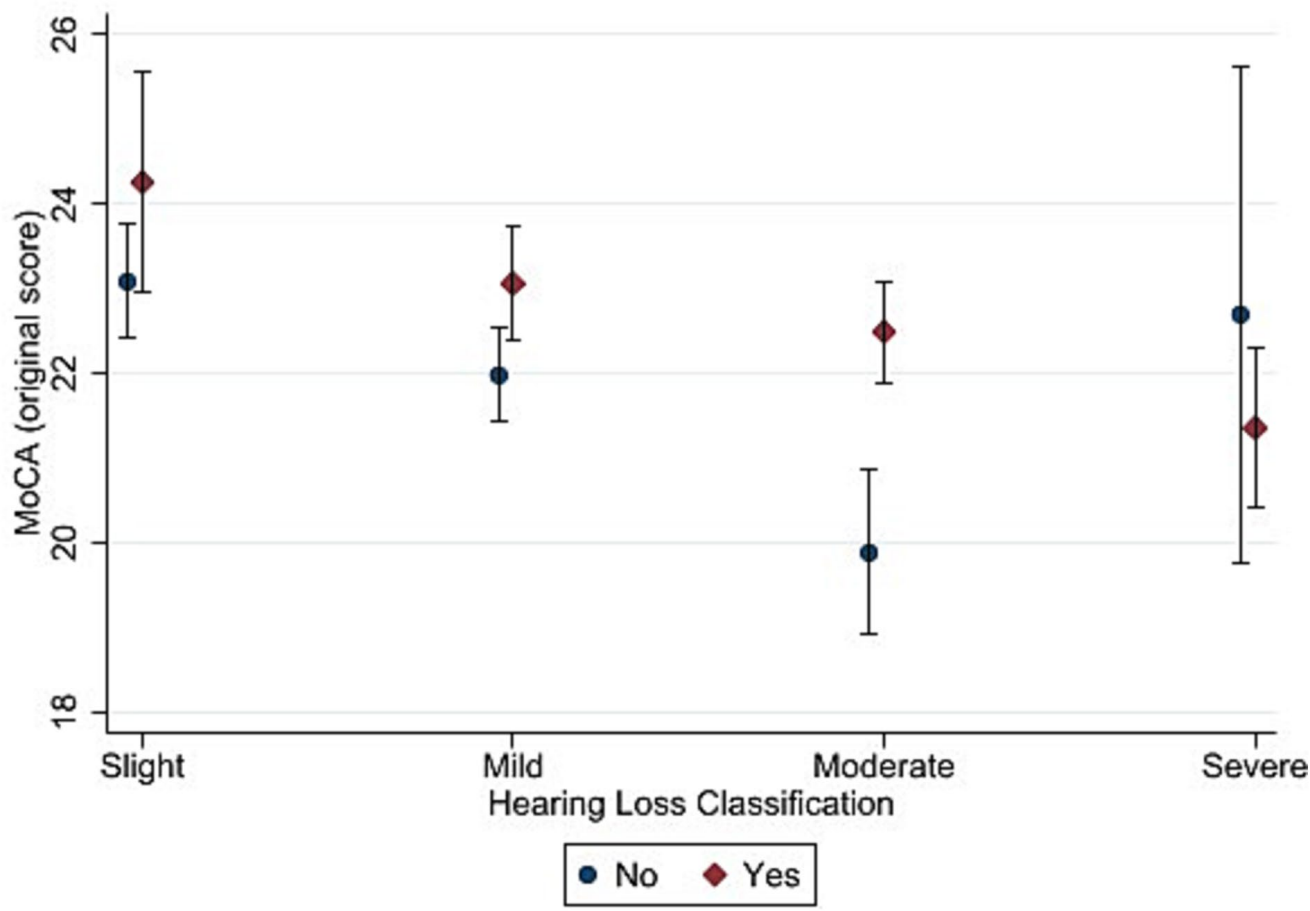
Estimated marginal means of the Montreal Cognitive Assessment (standard scoring) as a function of hearing status and hearing aid use. “Yes” in the figure refers to participants with hearing loss who used hearing aid and “No” refers to those who did not use hearing aid. Estimates are averaged over categories of age, education, and sex at the means of the covariates of heart attack, heart failure, cancer, stroke, diabetes, smoking, and hospital admission for head injury. The error bars indicate 95% confidence intervals.

**Figure 4:**
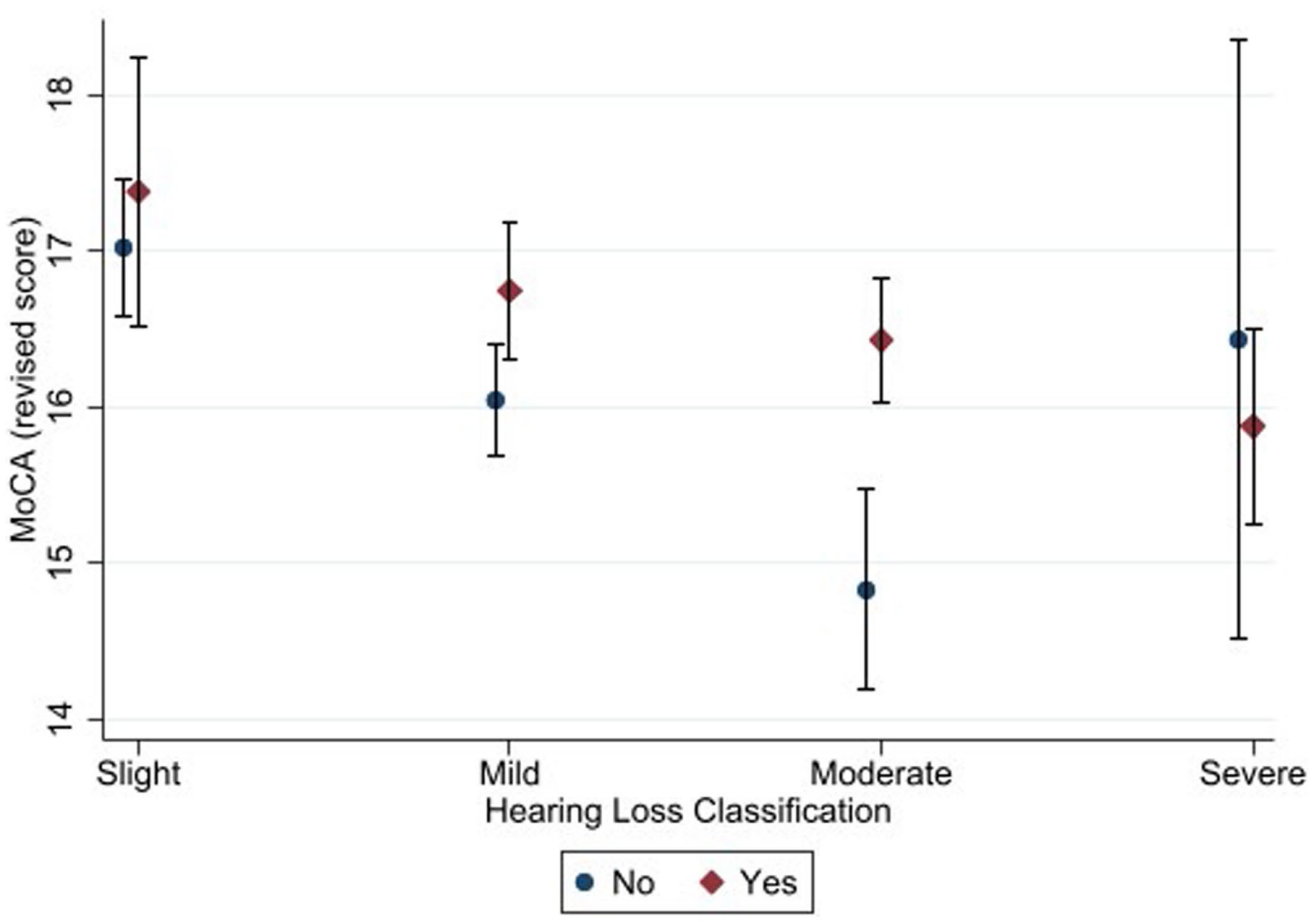
Estimated marginal means of the Montreal Cognitive Assessment (alternate scoring) as a function of hearing status and hearing aid use. “Yes” in the figure refers to participants with hearing loss who used hearing aid and “No” refers to those who did not use hearing aid. Estimates are averaged over categories of age, education, and sex at the means of the covariates of heart attack, heart failure, cancer, stroke, diabetes, smoking, and hospital admission for head injury. The error bars indicate 95% confidence intervals.

Figures 3, 4 depict the estimated marginal means for the standard and alternate scoring of the MoCA as a function of hearing status and hearing aid use in the hearing-impaired groups. For the standard scoring procedure, a five-way ANOVA showed a significant main effects of hearing status [*F* (3, 2079) = 7.22, *p* < 0.001, η^2^ = 0.011]. Bonferroni-adjusted pairwise comparisons showed that the slight hearing loss group (M *=* 23.6) achieved a higher score than the mild (M *=* 22.5), moderate (M *=* 21.3) and severe (M *=* 21.6) hearing loss groups. In addition, mild hearing loss group achieved higher score than moderate hearing loss group. The results also showed a significant main effect of hearing aid use [*F* (1, 2079) = 10.52, *p* < 0.001, η^2^ = 0.006]. Bonferroni-adjusted pairwise comparisons showed that hearing aid users (M = 22.7) achieved higher score than non-hearing-aid users (M = 21.7). The interaction between hearing status and hearing aid use was not significant [*F* (3, 2079) = 1.47, *p* = 0.221].

The pattern of results was similar for the alternate scoring procedure. The main effect of hearing status was significant [*F* (3, 2079) = 8.48, *p* < 0.001, η^2^ = 0.013]. Bonferroni-adjusted pairwise comparisons showed that the slight hearing loss group (M *=* 17.2) achieved a higher score than the mild (M *=* 16.4), moderate (M *=* 15.7) and severe (M *=* 16.0) hearing loss groups. In addition, mild hearing loss group achieved higher score than moderate hearing loss group.

The main effect of hearing aid use was also significant [*F* (1, 2079) = 12.77, *p* < 0.001, η^2^ = 0.007]. Bonferroni-adjusted pairwise comparisons showed that hearing aid users (M = 16.6) achieved higher score than non-hearing-aid users (*M* = 16.0). The interaction between hearing status and hearing aid use was not significant (*F* (3, 2079) = 1.08, *p* = 0.355).

## Discussion

This cross-sectional study showed that poorer hearing ability in older adults was associated with lower MoCA scores. These findings are in line with prior research showing worse MoCA performance for hearing-impaired individuals than for individuals with normal hearing (e.g., Dupuis et al., 2015; Lim and Loo, 2018; Fu et al., 2023). Pairwise comparisons showed that only participants with moderate and severe hearing loss had lower MoCA scores than people with normal hearing. These findings are in line with a study by Fu et al. (2023), which revealed that normal hearing, when comparing with moderate/severe hearing loss (and not mild hearing loss), was associated with better scores on the alternate version of the MoCA for hearing impairment (HI-MoCA). In addition, our findings add to the literature by showing a trend whereby individuals with severe hearing loss attained lower MoCA than those with less severe hearing loss. This finding is in line with Alattar et al. (2020), who in a 24-years longitudinal study, showed that hearing loss was associated with an accelerated decline in global cognitive function (measured with the Mini-Mental State Examination) and executive function (measured with the Trail-Making Test Part B) in older adults, and this cognitive decline was more evident among people with moderate/severe hearing loss than those with mild hearing loss.

One explanation for these findings might be that pure-tone audiometry (PTA) is cognitively demanding, especially for older adults, requiring the attention of listeners to detect tones at varying frequencies (see Heinrich et al., 2020). Castiglione et al. (2019) found a significant correlation between PTA and MoCA scores among older adults. We speculate that older adults with higher PTA thresholds (or more severe degrees of hearing loss) need to dedicate more cognitive resources to sound processing (than those with lower PTA thresholds or less severe hearing loss) to extract meaning from a talker in a cognitive test. Fewer resources are therefore available for the functions of storage and retrieval of memory items in the MoCA test (see the Ease of Language Understanding model; Rönnberg et al., 2021, 2022). Consequently, people with more severe hearing loss perform worse on the MoCA than those with normal hearing or less severe hearing loss.

The question that arises is that if auditory acuity is the main reason for poor MoCA performance in older adults with hearing loss, why is the pattern of results not opposite in the alternate MoCA scoring for people with hearing loss (after removing those MoCA items that rely on auditory function)? To answer this question, we refer to those studies which indicate that hearing loss *per se* adversely affects cognitive function in people with hearing loss (Andersson and Lyxell, 1998; Andersson, 2002; Classon et al., 2014). For example, Classon et al. (2014) reported that moderate-to-severe hearing loss can lead to deterioration in phonological representations in semantic long-term memory, which presumably has a negative impact when searching for lexical items or making semantic categorization decisions that supposedly are needed for appropriate performance in a cognitive task. Dupuis et al. (2015), in their study, also showed that even after eliminating the MoCA items that rely on the auditory function, people with hearing loss performed worse in the MoCA than those with normal hearing.

In fact, studies have shown that hearing loss is independently associated with atrophy or structural changes in brain areas involved in cognitive function, like the hippocampus (Belkhiria et al., 2019; Chen et al., 2020; Croll et al., 2020; Xing et al., 2020; Aoki et al., 2021). For instance, Aoki et al. (2021) revealed atrophy of the hippocampus in participants with moderate/severe hearing loss relative to participants with normal hearing or mild hearing loss. Croll et al. (2020) reported that hearing loss is linked to lower microstructural integrity in brain areas involved in cognitive processes. Future studies are needed to determine whether poor auditory function or hearing loss *per se* (or a combination of both) are the main cause of poor cognitive function in people with hearing loss.

In this study, the prevalence of hearing aid use among older adults with various degrees of hearing loss was as follows: slight hearing loss 4.3%, mild hearing loss 22.1%, moderate hearing loss 61.7%, and severe hearing loss 78.3%. Reed et al. (2023), in a national cohort study conducted in the USA, reported the prevalence of hearing-aid use among older Americans (aged 71+ years) with various degrees of hearing loss as: 14.4% in mild hearing loss, 45.3% in moderate hearing loss, and 67.9% in severe hearing loss. It seems that hearing aid use is more common in our study than in that by Reed et al. (2023).

Our results showed an association between hearing aid use and MoCA performance, and hearing-aid users achieved higher MoCA scores than non-users. Our findings are in line with Glick and Sharma (2020), who showed hearing aid use was associated with better MoCA performance, and hearing aid use could reverse neural changes caused by hearing loss. Naylor et al. (2022) revealed that although hearing aid use reduced the risk of dementia in people with hearing loss, pre-existing dementia was associated with reduced persistency in use of hearing aid among people with hearing loss. In fact, Naylor et al. (2022) showed that the cognitive function of people with hearing loss plays a critical role in persistent use of hearing aid. We speculate that cognitively healthy participants are more likely to wear their hearing aids than those suffering from dementia, which subsequently would lead to better performance in the MoCA test. Our finding can be interpreted as hearing aid can safeguard against cognitive decline in people with hearing loss or persons with greater function were persistent hearing-aid users. Taken together and based on our result that hearing-aid users achieved higher MoCA scores than non-users, we suggest that access to hearing aid is important for cognitive function in people with hearing loss.

Finally, the pattern of results between the two different scoring methods (standard scoring and alternate scoring) was highly similar. This finding is in line with a study by Shen et al. (2020), which examined the extent to which the test administration conditions (auditory amplified, auditory unamplified, and visual) affected the MoCA performance of older adults with hearing loss. Their results showed that neither amplification nor test modality influenced participants’ performance in the MoCA. In addition, our results are in line with a study by Parada et al. (2020), which compared HI-MoCA with the standard version of the MoCA among postlingually deafened cochlear implant users. No significant difference was observed in participants’ performance between the HI-MoCA and standard MoCA. Similarly, Lin et al. (2017) compared a visual adaptation of the MoCA (HI-MoCA; using PowerPoint presentation software) with the standard version of the MoCA among people with severe hearing loss. The results showed no significant clinical differences between the HI-MoCA and the standard MoCA. Al-Yawer et al. (2019) also revealed that deleting some items of the MoCA adversely affected the sensitivity and specificity of the MoCA for the evaluation of cognitive function.

The current research is strengthened by the large sample of participants, within a population-health study, which gave us enough power to detect small differences between groups with different hearing statuses in terms of their performance in the MoCA. The controlling of confounders and health covariates likely to have biased the results was another strength.

The current study has some limitations that should be taken into consideration. First, the sample in this study included both cognitively healthy participants and participants with mild cognitive impairment (MCI) or dementia. As hearing loss and cognitive impairment often co-occur, it can not be excluded that there are more people with MCI or dementia among participants with moderate or severe degrees of hearing loss that performed worse on both standard and alternate scoring of the MoCA test. Excluding participants with MCI or dementia may have resulted in a different impact of hearing loss performance on both standard and alternate scoring of the MoCA. Second, the duration of hearing aid use and duration of hearing loss – important variables when considering associations between hearing loss, hearing aid use and performance in the MoCA – were not collected in the HUNT4 study. Third, the current study is a cross-sectional study that limits causal links between participants’ hearing status, hearing aid use and MoCA performance. Future studies are required to determine the causal links between hearing loss and hearing aid use and MoCA performance. More specifically, longitudinal studies and preferable randomized trials, are needed to evaluate how hearing aid use can safeguard against the incidence of cognitive decline and dementia. Lin et al. (2023), in a randomized controlled trial, evaluated the effectiveness of hearing aid intervention on cognitive decline among older adults with hearing loss. The experimental group received audiological counselling and provision of hearing aids, while the control group only received health education on chronic disease prevention. A three-year follow-up assessment showed no effect of hearing aid intervention on cognitive decline. Nonetheless, sensitivity analysis showed that hearing aid intervention might reduce cognitive decline among older adults with hearing loss who are at more risk of cognitive decline. Fourth, the participation rate in the HUNT4 70+ study was around 51%, which may restrict the generalizability of the findings. Fifth, and importantly, the effect sizes for the main effect of hearing status were small. This may indicate that there are factors, other than participants’ hearing status, that affected performance in the MoCA in this study of older adults.

## Conclusion

This cross-sectional study revealed an association between hearing status and MoCA scores in older adults. Only those individuals with moderate or severe hearing loss performed worse in the MoCA than individuals with normal hearing. An association between hearing aid use and MoCA score was observed, and hearing aid users achieved higher MoCA scores than non-users. Nevertheless, randomized trials are needed to evaluate how hearing aid use can safeguard against the incidence of cognitive decline and dementia. The pattern of results between the standard scoring of the MoCA and the alternate version for hearing loss was similar.

## Data availability statement

The datasets presented in this article are not readily available because parts of HUNT study in Norway was used for the purpose of this submission. Anyone interested to the data for this submission should contact PIs of HUNT study (https://www.ntnu.edu/hunt). Requests to access the datasets should be directed to https://www.ntnu.edu/hunt.

## Ethics statement

The studies involving humans were approved by the regional committee for medical and health research ethics (ID Number: 23178 Hørsel). The studies were conducted in accordance with the local legislation and institutional requirements. The participants provided their written informed consent to participate in this study.

## Author contributions

SM: Conceptualization, Data curation, Formal analysis, Investigation, Methodology, Writing – original draft. BE: Conceptualization, Data curation, Formal analysis, Funding acquisition, Investigation, Methodology, Project administration, Visualization, Writing – original draft. AJ: Writing – original draft. GS: Conceptualization, Funding acquisition, Project administration, Writing – original draft. LA: Funding acquisition, Project administration, Writing – original draft. GH: Conceptualization, Writing – original draft.
